# Supplementary material for: Secular Trends in Systemic Sclerosis Mortality in the United States from 1981 to 2020
Source: Int J Environ Res Public Health. 2022 Nov 16;19(22):15088. doi: 10.3390/ijerph192215088 (PMC9690027; doi:10.3390/ijerph192215088)
Supplement: Supplementary file 1 [file ijerph-19-15088-s001.zip › ijerph-1932084-supplementary.pdf]

Supplementary Figure S1. The columns of cause of death on United State standard death certificate.

CAUSE OF DEATH (See instructions and examples)

32. **PART I.** Enter the chain of events--diseases, injuries, or complications--that directly caused the death. DO NOT enter terminal events such as cardiac arrest, respiratory arrest, or ventricular fibrillation without showing the etiology. DO NOT ABBREVIATE. Enter only one cause on a line. Add additional lines if necessary.

		Approximate interval: Onset to death
IMMEDIATE CAUSE (Final disease or condition **--------->** resulting in death)	a.____________________	________
	Due to (or as a consequence of):	
Sequentially list conditions, if any, leading to the cause listed on line a. Enter the **UNDERLYING CAUSE** (disease or injury that initiated the events resulting in death) **LAST**	b.____________________	________
	Due to (or as a consequence of):	
	c.____________________	________
	Due to (or as a consequence of):	
	d.____________________	________

Supplementary Table S1. The number of deaths and age-standardized mortality rate (ASMR) based on the underlying cause of death, 1981–2020.

	**(1)1981–1990**			**(2)1991–2000**			**(3)2001–2010**			**(4)2011–2020**		
**Age**	**No**	**%**	**ASMR**	**No**	**%**	**ASMR**	**No**	**%**	**ASMR**	**No**	**%**	**ASMR**
Both sexes												
Overall*	8067	100.0	3.6	11379	100.0	4.3	13292	100.0	4.3	11934	100.0	3.2
0–44	1065	13.2	0.6	1272	11.2	0.7	1261	9.5	0.7	859	7.2	0.5
45–64	3222	39.9	7.2	4000	35.2	7.4	4973	37.4	6.7	4040	33.9	4.8
65–74	2316	28.7	13.6	3370	29.6	18.1	3354	25.2	17.2	3401	28.5	12.2
≥75	1464	18.1	12.5	2737	24.1	18.1	3704	27.9	20.8	3634	30.5	17.6
Men												
Overall*	1914	100.0	1.9	2302	100.0	2.0	2604	100.0	1.8	2294	100.0	1.3
0–44	237	12.4	0.3	296	12.9	0.3	329	12.6	0.3	222	9.7	0.2
45–64	866	45.2	4.0	956	41.5	3.6	1260	48.4	3.5	974	42.5	2.4
65–74	539	28.2	7.3	633	27.5	7.6	586	22.5	6.5	656	28.6	5.0
≥75	272	14.2	6.6	417	18.1	7.7	429	16.5	6.3	442	19.3	5.2
Women												
Overall*	6153	100.0	5.0	9077	100.0	6.3	10688	100.0	6.3	9640	100.0	4.8
0–44	828	13.5	1.0	976	10.8	1.1	932	8.7	1.0	637	6.6	0.7
45–64	2356	38.3	10.0	3044	33.5	10.9	3713	34.7	9.8	3066	31.8	7.2
65–74	1777	28.9	18.5	2737	30.2	26.5	2768	25.9	26.3	2745	28.5	18.5
≥75	1192	19.4	15.7	2320	25.6	24.0	3275	30.6	29.8	3192	33.1	26.0

*Mortality rate was age-adjusted using the United States population in 2000 as standard population.

Supplementary Table S2. The number of deaths and age-standardized mortality rate (ASMR) based on the multiple causes of death, 1981–2020.

	**(1)1981–1990**			**(2)1991–2000**			**(3)2001–2010**			**(4)2011–2020**		
**Age**	**No**	%	**ASMR**	**No**	%	**ASMR**	**No**	%	**ASMR**	**No**	%	**ASMR**
Both sexes												
Overall*	12896	100.0	5.8	17665	100.0	6.7	18733	100.0	6.0	16965	100.0	4.5
0–44	1411	10.9	0.9	1695	9.6	0.9	1587	8.5	0.9	1106	6.5	0.6
45–64	4899	38.0	10.9	5786	32.8	10.7	6647	35.5	9.0	5572	32.8	6.7
65–74	3863	30.0	22.7	5312	30.1	28.6	4826	25.8	24.7	4835	28.5	17.4
≥75	2723	21.1	23.2	4872	27.6	32.2	5673	30.3	31.9	5452	32.1	26.3
Men												
Overall*	3025	100.0	3.1	3547	100.0	3.1	3624	100.0	2.5	3229	100.0	1.9
0–44	323	10.7	0.4	402	11.3	0.4	413	11.4	0.4	290	9.0	0.3
45–64	1315	43.5	6.1	1344	37.9	5.1	1666	46.0	4.6	1366	42.3	3.4
65–74	897	29.7	12.1	1042	29.4	12.6	851	23.5	9.5	914	28.3	7.0
≥75	490	16.2	11.9	759	21.4	13.9	694	19.2	10.2	659	20.4	7.8
Women												
Overall*	9871	100.0	8.0	14118	100.0	9.7	15109	100.0	8.9	13736	100.0	6.8
0–44	1088	11.0	1.3	1293	9.2	1.5	1174	7.8	1.3	816	5.9	0.9
45–64	3584	36.3	15.3	4442	31.5	15.9	4981	33.0	13.2	4206	30.6	9.8
65–74	2966	30.0	30.9	4270	30.2	41.4	3975	26.3	37.7	3921	28.5	26.4
≥75	2233	22.6	29.4	4113	29.1	42.5	4979	33.0	45.2	4793	34.9	39.0

*Mortality rate was age-adjusted using the United States population in 2000 as standard population.

Supplementary Table S3. Annual percent changes (APC) in age-standardized mortality rate of systemic sclerosis according to joinpoint analysis based on the underlying cause of death (UCD) and multiple causes of death (MCD) data, 1981–2020.

	**Trend 1**			**Trend 2**			**Trend 3**			**Trend 4**		
**Age**	**Year**	**APC**	***p***	**Year**	**APC**	***p***	**Year**	**APC**	***p***	**Year**	**APC**	***p***
UCD, men												
Overall	1981–2002	0.4	0.133	2002–2020	−3.3	<0.001						
0–44	1981–2002	1.7	0.019	2002–2020	−3.5	<0.001						
45–64	1981–2005	−0.4	0.251	2005–2020	−4.3	<0.001						
65–74	1981–2000	0.8	0.245	2000–2020	−3	<0.001						
≥75	1981–1995	2.7	0.053	1995–2020	−2.2	<0.001						
UCD, women												
Overall	1981–1986	−0.9	0.471	1986–1999	3.1	<0.001	1999–2013	−1.9	<0.001	2013–2020	−4.4	<0.001
0–44	1981–2001	1	0.018	2001–2020	−3.6	<0.001						
45–64	1981–1986	−4	0.036	1986–1997	2.1	0.002	1997–2007	−1.6	0.014	2007–2020	−3.8	<0.001
65–74	1981–2000	3.8	<0.001	2000–2020	−3.3	<0.001						
≥75	1981–2001	4.5	<0.001	2001–2013	−0.4	0.493	2013–2020	−3.4	0.004			
MCD, men												
Overall	1981–2000	−0.1	0.687	2000–2020	−3.2	<0.001						
0–44	1981–2002	1.3	0.051	2002–2020	−3.5	<0.001						
45–64	1981–2004	−1.1	<0.001	2004–2020	−3.3	<0.001						
65–74	1981–1997	0.7	0.367	1997–2020	−3.2	<0.001						
≥75	1981–1993	3.3	0.01	1993–2020	−2.9	<0.001						
MCD, women												
Overall	1981–1999	2.1	<0.001	1999–2020	−2.6	<0.001						
0–44	1981–2000	1.1	0.011	2000–2020	−3.5	<0.001						
45–64	1981–1998	0.5	0.041	1998–2020	−2.9	<0.001						
65–74	1981–1999	3.1	<0.001	1999–2020	−3.3	<0.001						
≥75	1981–1999	4	<0.001	1999–2020	−1.3	<0.001						

Supplementary Table S4. The 5-year mortality rate per age group of 5 years. (A) underlying cause of death, (B) multiple causes of death.

(A)

Age	Period 1980–1984	1985–1989	1990–1994	1995–1999	2000–2004	2005–2009	2010–2014	2015–2019
20–24	0.3	0.3	0.3	0.3	0.4	0.3	0.3	0.2
25–29	0.6	0.6	0.5	0.6	0.6	0.6	0.4	0.4
30–34	1.1	0.8	0.8	0.8	0.8	0.8	0.8	0.6
35–39	1.7	1.3	1.6	1.5	1.7	1.4	1.1	1.1
40–44	2.8	2.9	2.4	2.5	2.9	2.6	1.8	1.5
45–49	4.5	3.8	4.0	4.5	4.1	3.8	2.8	2.4
50–54	5.9	5.6	5.8	6.6	6.0	5.6	4.7	3.7
55–59	8.7	7.6	8.7	9.1	8.6	8.0	6.0	4.9
60–64	11.1	10.7	11.7	13.4	13.4	10.7	9.3	6.9
65–69	12.2	13.0	14.8	17.0	16.3	14.8	12.7	9.9
70–74	12.9	15.0	18.6	20.9	21.8	18.7	16.7	13.8
75–79	12.4	16.7	18.2	20.5	23.6	23.0	20.5	17.0
80–84	9.3	12.5	14.7	20.7	23.6	22.1	21.0	18.9
≥85	6.3	7.7	11.5	13.8	14.3	16.3	16.5	14.8

(B)

Age	Period 1980–1984	1985–1989	1990–1994	1995–1999	2000–2004	2005–2009	2010–2014	2015–2019
20–24	0.4	0.4	0.4	0.3	0.5	0.3	0.3	0.2
25–29	0.7	0.7	0.6	0.8	0.8	0.7	0.6	0.5
30–34	1.3	1.1	1.1	1.2	1.0	1.1	1.0	0.8
35–39	2.2	1.7	2.0	2.0	2.1	1.7	1.4	1.3
40–44	3.8	4.1	3.5	3.5	3.8	3.2	2.3	2.0
45–49	6.1	5.4	5.4	6.1	5.3	5.2	3.7	3.2
50–54	8.2	8.5	8.7	9.1	8.2	7.0	6.0	5.2
55–59	13.5	12.0	13.3	13.1	12.3	10.4	8.3	7.0
60–64	17.0	17.8	18.2	19.8	18.7	14.2	12.2	10.0
65–69	19.9	21.7	24.4	25.7	24.0	20.3	16.9	14.0
70–74	21.8	25.7	29.7	33.3	32.7	26.8	24.2	19.6
75–79	22.4	28.7	32.1	34.8	36.3	34.0	28.4	24.1
80–84	19.5	24.1	28.9	37.2	36.8	33.7	30.8	28.2
≥85	14.1	16.4	22.6	27.0	24.4	25.3	26.7	24.3
